# Iodides and Salicylates

**Published:** 1890-11-15

**Authors:** 


### THE PRACTITIONER'S MIRROR AND RETROSPECT.

### MEDICINE.

### IODIDES AND SALICYLATES.

The investigations of Rosenbach and Pohl on the different and, in some respects, complementary actions of the iodides and the salicylates, the value of which in the treatment of rheumatism is well known, and which in chronic cases act well in combination, appear to be of considerable practical interest.

Salicyl compounds taken into the stomach pass into the urine, and into the fluids of serous and synovial sacks. But they are not found in the saliva, the bile, or even in the contents of the bowel. Iodine compounds, on the other hand, given internally or by subcutaneous injection, rapidly pass into the saliva and urine and are found in *morbid transudations* into the skin, abdomen, and pleura, but they are never to be met with in serous or purulent exudations, nor in the synovial fluids, whether in health or disease. Both iodides and salicylates injected into joints or serous cavities when transudations, or serous or purulent exudations are present, are soon afterwards to be detected in the urine.

But the essential difference in their respective behaviours, the therapeutic bearing of which is evident, is that salicyl, however administered, is found in all serous and synovial cavities, whereas iodine, at least when given by the mouth and in the usual doses, passes into transudations only, and never into synovial sacs, whether normal or inflamed.

